# Anatomy and surgical planes of the pelvic cavity based on the peritoneum retreat theory

**DOI:** 10.3389/fsurg.2026.1841593

**Published:** 2026-05-21

**Authors:** Shicai Chen, Guohua Yang, Nanrong Yu

**Affiliations:** Department of Gastrointestinal Tumor Surgery, Affiliated Cancer Hospital and Institute of Guangzhou Medical University, Guangzhou, China

**Keywords:** denonvilliers' fascia, extraperitoneal fascia, peritoneum fusion theory, peritoneum retreat theory, rectosacral fascia, transversalis fascia

## Abstract

**Background:**

Several studies have described the structure of the pelvic cavity; however, there is still confusion about the optimal dissection plane of rectal surgery. The aim of this study was to clarify the anatomical structures of the pelvic cavity and identify the correct surgical plane for rectal cancer based on the peritoneum retreat theory.

**Methods:**

Surgical videos of 29 male patients who underwent laparoscopic operations for rectal cancer were reviewed to identify the anatomical structures of the mesorectum and its surrounding fasciae. Schematic diagrams and sectional illustrations of the pelvic cavity were generated to illustrate its anatomical structures.

**Results:**

The general anatomy of the pelvic cavity could be divided into three layers (pelvic wall, urogenital system, and rectum) and two interlayers (transversalis fascia and extraperitoneal fascia). The urogenital system layer was an independent viaduct-like structure sandwiched between the pelvic wall and the rectum. The rectosacral fascia was the lower part of the urogenital system layer and was separated from the fascia propria of the rectum by the junction of transversalis fascia and extraperitoneal fascia at the level of S3/4 vertebrae. Posterolateral to the upper rectum, the presacral fascia and posterior renal fascia defined the presacral space, while the anterior renal fascia (also called Gerota's fascia/prehypogastric nerve fascia) and the fascia propria of the rectum defined the retrorectal space. Behind the lower rectum, the presacral fascia and the fascia propria of the rectum defined the supralevator space. Denonvilliers’ fascia in front of the rectum was continuous with the pre-hypogastric nerve fascia. Denonvilliers’ fascia and the fascia propria of the rectum in the front defined the prerectal space.

**Conclusion:**

The rectosacral fascia is continuous with the urogenital system layer and not directly connected to the posterior rectal wall. The retrorectal, supralevator, and prerectal spaces form the correct surgical plane for mesenteric-based rectal surgery by operating within the extraperitoneal fascia.

## Introduction

In recent years, mesenteric-based surgery has received more attention because a correct anatomical plane results in safe and reproducible surgery and achieves an optimal oncological outcome (1). Heald et al. showed that total mesorectal excision (TME) for rectal cancer relied on dissection through the optimal surgical plane called the “holy plane” (2, 3). The “holy plane” was presented as an avascular interface between viscus and soma. It was suggested that there was usually no ‘surgical plane’ behind Denonvilliers’ fascia, and TME had focused on dissection of the plane between the seminal vesicles and the anterior surface of Denonvilliers’ fascia (3, 4). However, the anatomy of the pelvic fasciae surrounding the rectum remains unclear, even though several attempts have been made to describe the structures of the pelvic cavity anatomically (5–12).

The mesentery and Toldt's fascia form a continuous structure that extends from the origin of the mesenteric organ (at the superior mesenteric artery) to the anorectal junction (1, 13). As a result of the mesenteric attachments, Toldt's fascia has always been considered as a fusion fascia formed by two layers of the visceral peritoneum, and the mesocolon is separated from the posterior abdominal wall by this connective tissue (14, 15). Several anatomical proposals for mesenteric-based surgery have been put forward in recent years based on the peritoneum fusion theory (1, 16). However, current research suggests that there may be several complex and contradictory issues to resolve (17). We previously proposed the peritoneum retreat theory, demonstrating that there is no fusion fascia in the abdomen due to retreat of the visceral peritoneum, and all of the fasciae surrounding the mesentery are extraperitoneal (17–19). This study aimed to clarify the anatomical structures of the pelvic cavity and the correct surgical planes for mesenteric-based rectal surgery based on the peritoneum retreat theory.

## Methods

### Surgical video selection and observation

Surgical videos of 29 male patients who underwent laparoscopic surgery for rectal cancer at the Affiliated Cancer Hospital and Institute of Guangzhou Medical University were reviewed to identify anatomical structures of the mesorectum and its surrounding fasciae. The presence of Denonvilliers’ fascia in females is still unclear (20, 21); therefore, only male rectal cancer patients were included in this study to avoid controversy. The patients’ age ranged from 25 to 76 years. No evidence of any other prior abdominal disease or surgery was found in these patients. Preoperative computed tomography (CT) or magnetic resonance imaging (MRI) showed that the clinical stage of the tumors was cT1-2N0M0. The patients were evaluated as unsuitable for endoscopic resection, and none of them had received preoperative chemoradiotherapy, targeted therapy, or immunotherapy. All these operations followed the principles of TME. In this study, the fasciae and spaces between adjacent tissues and organs in the pelvic cavity were observed and confirmed by different surgeons. If there was any ambiguity, the same conclusion would be reached after discussion.

### Schematic reconstructions and dynamic models of the pelvic cavity

We previously demonstrated that the general anatomical structures of the abdomen can be divided into three layers (abdominal wall, urogenital system, and digestive system) and two interlayers (transversalis fascia and extraperitoneal fascia) (17). Schematic diagrams and sectional illustrations of the pelvic cavity were generated to show its anatomical structures. Flash 8 and Adobe After Effects animation software was applied to establish schematic reconstructions and dynamic models. The study protocol was approved by the Affiliated Cancer Hospital and Institute of Guangzhou Medical University Ethical Committee. Written informed consent was obtained from all the selected patients, and ethical approval was granted.

## Results

### General anatomy of the pelvic cavity: perirectal fasciae and spaces

**Figure 1:**
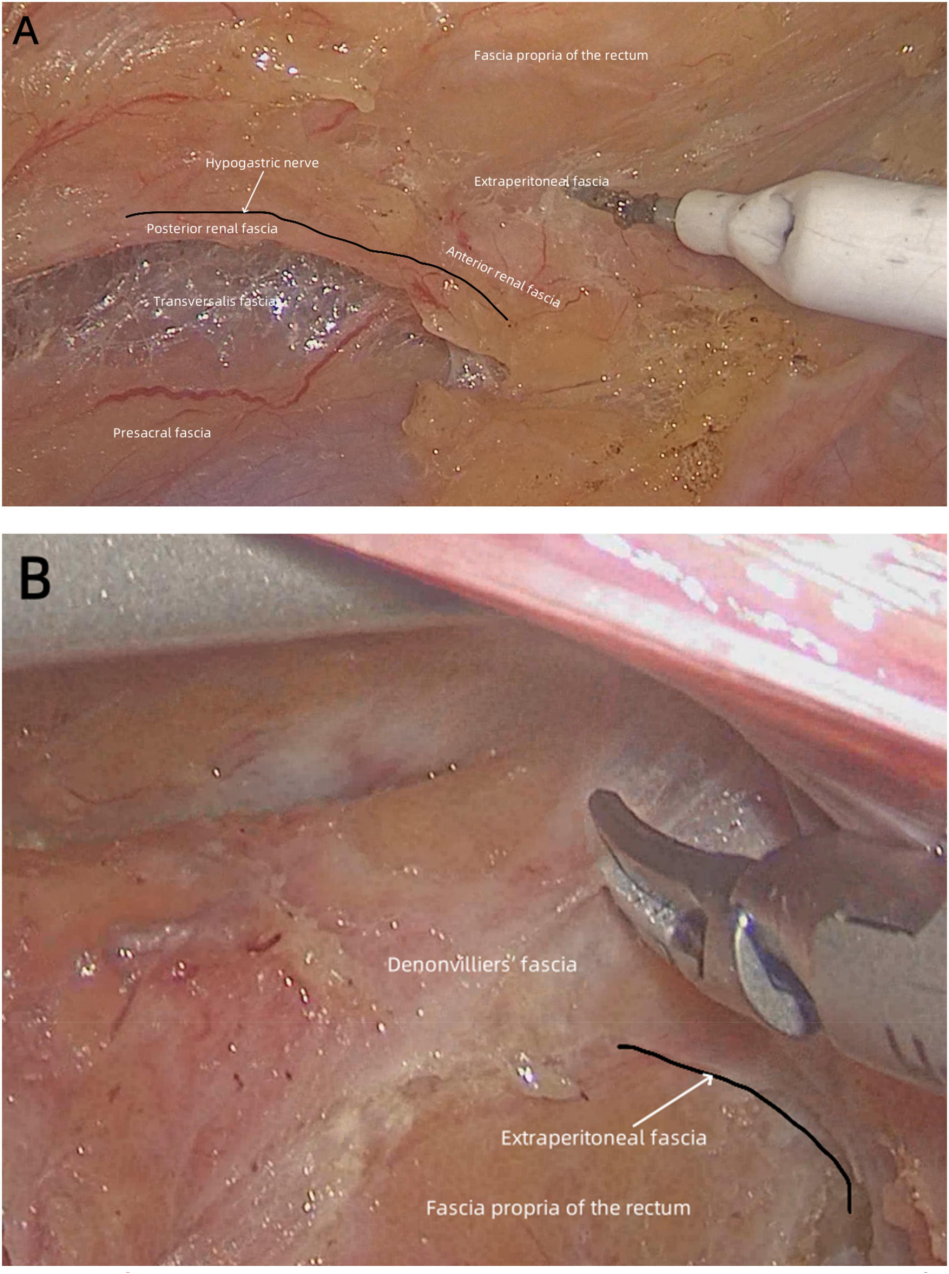
(case 1) **(A)** the urogenital system is located between the transversalis fascia and extraperitoneal fascia, by which it is separated from the pelvic wall behind and from the rectum ahead, respectively. Behind the upper rectum, the presacral fascia and the posterior renal fascia define the presacral space, while the anterior renal fascia and the fascia propria of the rectum define the retrorectal space. **(B)** In the front of the rectal wall, Denonvilliers’ fascia and the fascia propria of the anterior rectum define the prerectal space, which is occupied by the extraperitoneal fascia.

**Figure 2:**
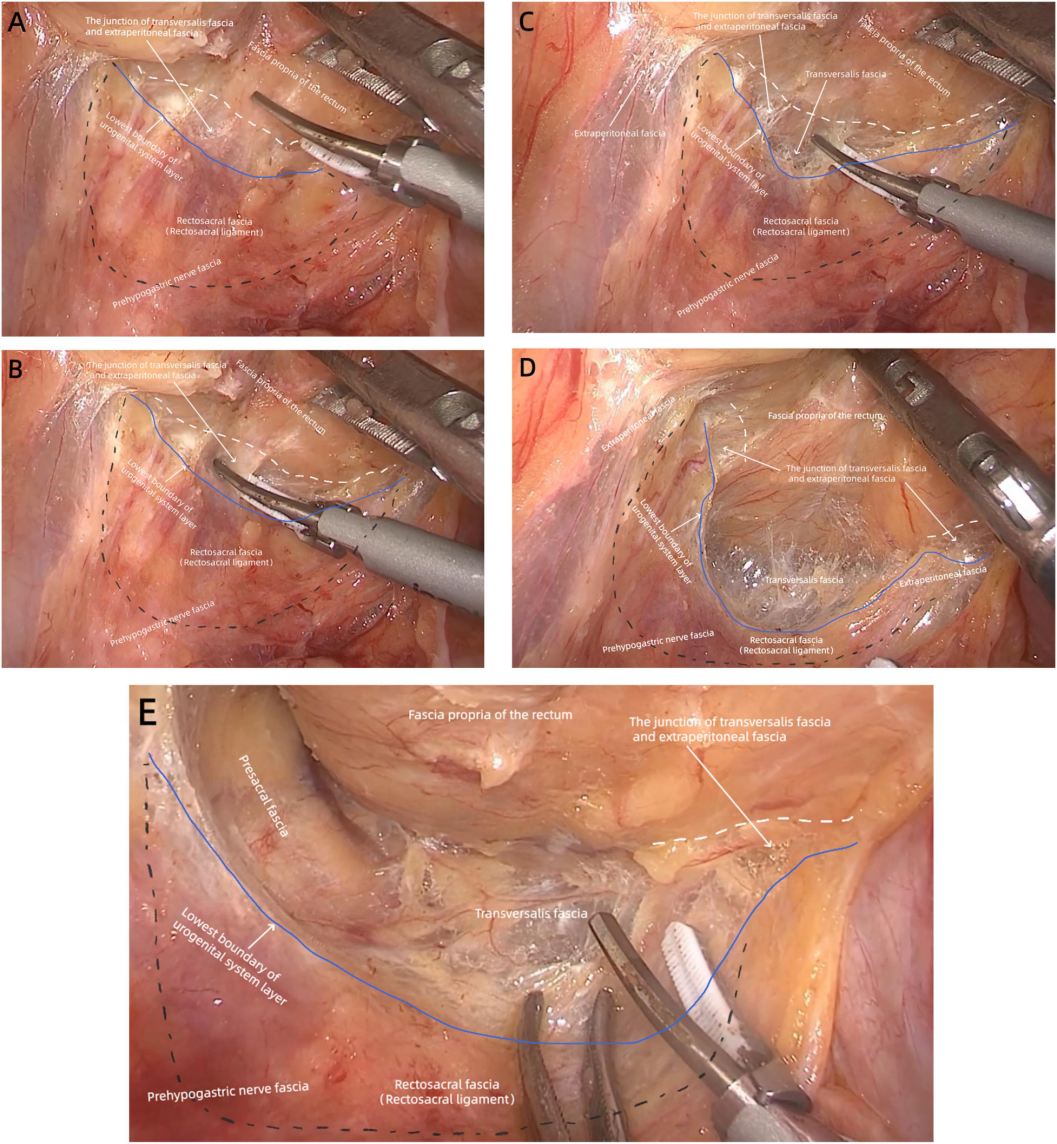
(case 2) **(A)** the prehypogastric nerve fascia covers the front of the urogenital system layer. The lowest boundary of the urogenital system layer (blue solid line) is separated from the fascia propria of the rectum (white dotted line) by the junction of transversalis fascia and extraperitoneal fascia at the S3/4 vertebral level. The rectosacral fascia/rectosacral ligament (black dotted area) is the lower part of the urogenital system layer and not directly connected to the posterior rectal wall. **(B)** The junction of the transversalis fascia and extraperitoneal fascia is severed to expose the supralevator space. **(C,D)** The junction of the transversalis fascia and extraperitoneal fascia is severed and the transversalis fascia within the supralevator space is exposed. **(E)** The transversalis fascia within the supralevator space is exposed and the supralevator space, which is the continuation of the presacral space, is formed by the presacral fascia and the fascia propria of the lower rectum.

**Figure 3:**
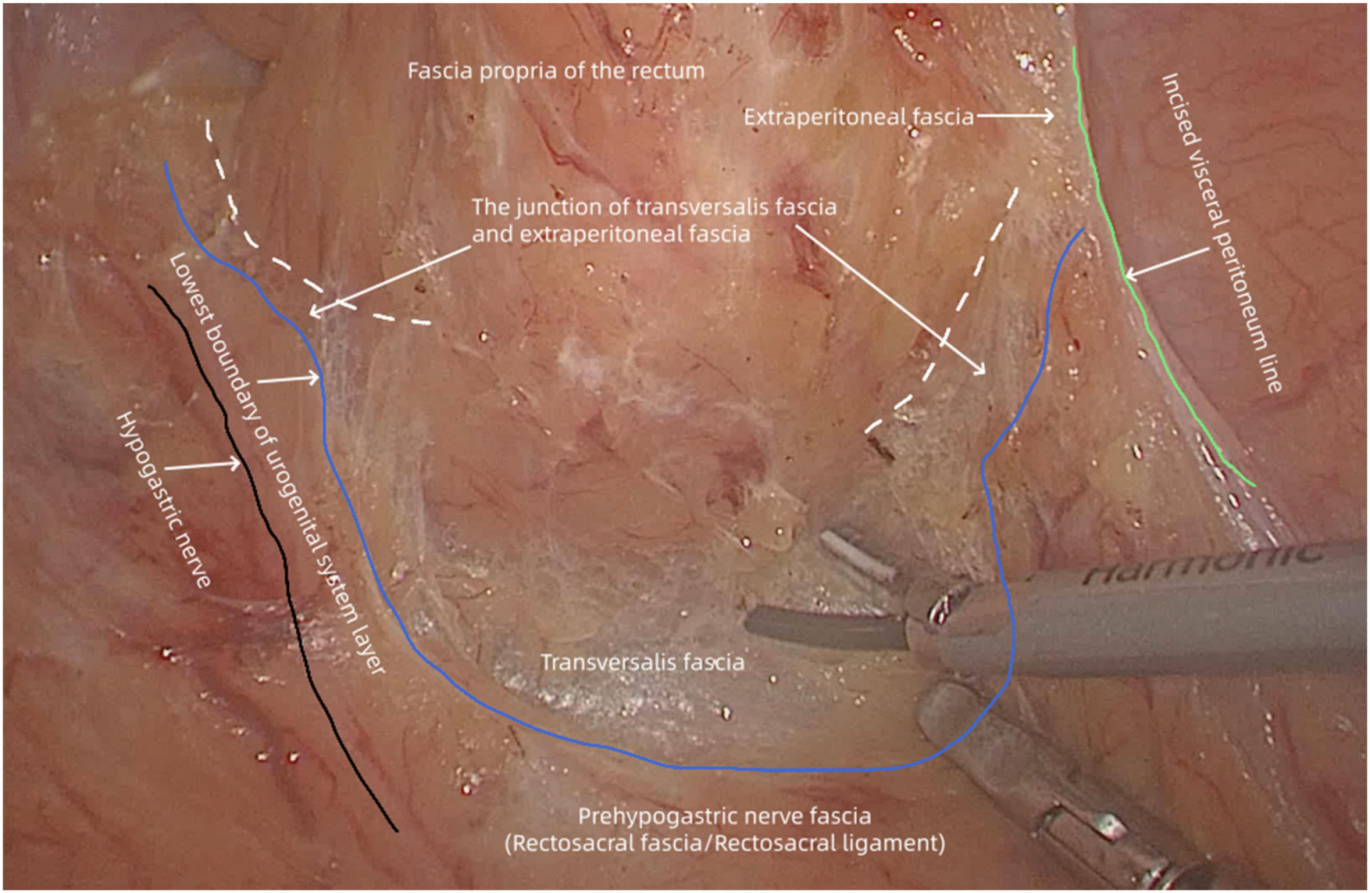
(case 3) the lowest boundary of the urogenital system layer (blue solid line) posterolateral to the rectum divides the rectum into the upper and lower sections. The extraperitoneal fascia is within the retrorectal space behind the upper rectum, while the transversalis fascia is within the supralevator space behind the lower rectum. The rectosacral fascia/rectosacral ligament are the lower part of the urogenital system layer and are separated from the fascia propria of the rectum by the junction of the transversalis fascia and extraperitoneal fascia.

**Figure 4:**
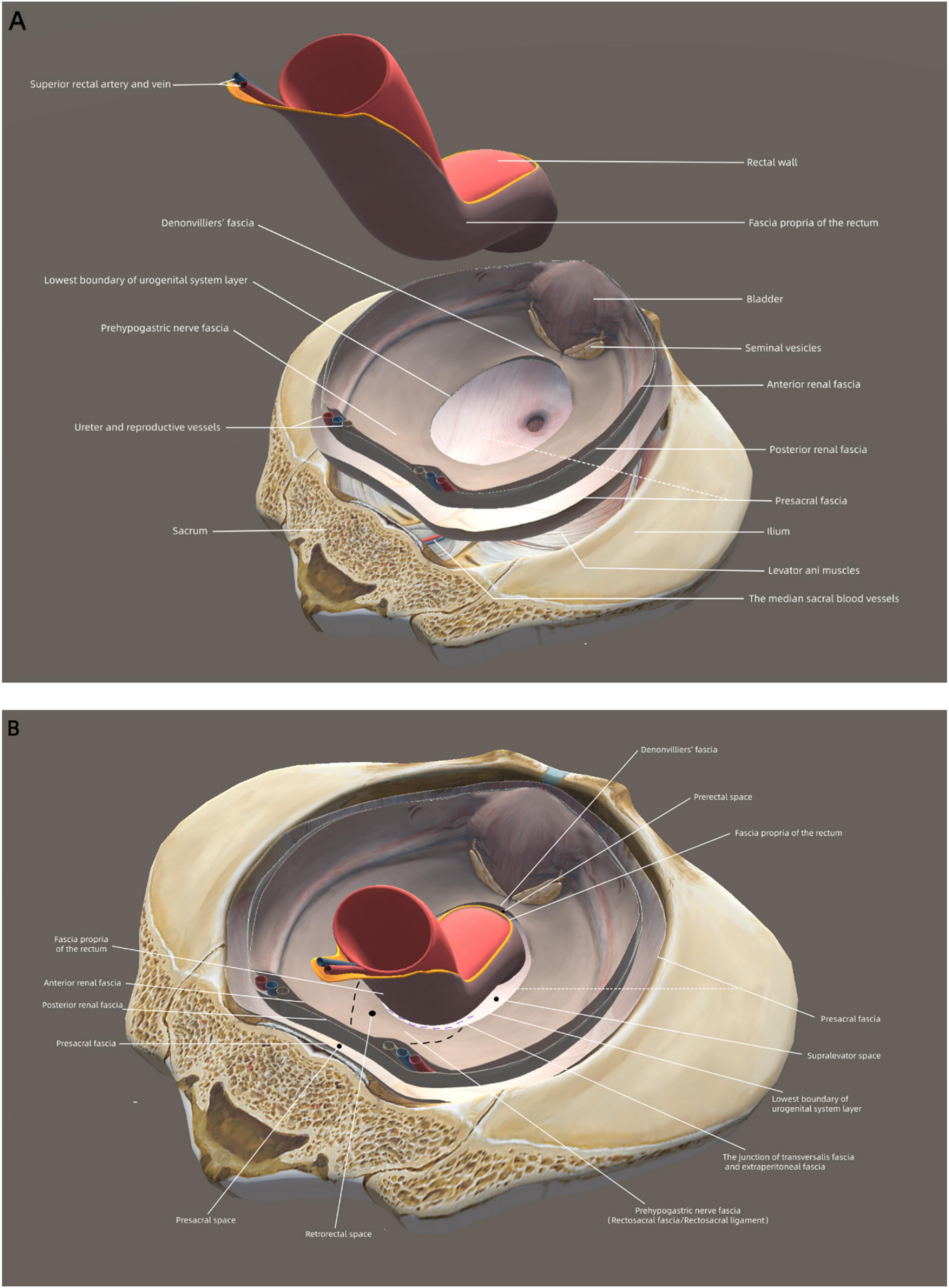
Schematic diagrams and sectional illustrations of the pelvic cavity based on the peritoneum retreat theory. **(A)** The urogenital system layer is an independent viaduct-like structure sandwiched between the pelvic wall and the rectum. The anterior renal fascia and the posterior renal fascia envelop the urogenital system layer to form a urogenital–hypogastric sheath, carrying the hypogastric nerves, ureter, and genital vessels downward to the pelvis. Denonvilliers’ fascia is continuous with the anterior renal fascia (prehypogastric nerve fascia). **(B)** In the posterior space of the upper rectum, the presacral fascia and the posterior renal fascia define the presacral space, while the anterior renal fascia and the fascia propria of the rectum define the retrorectal space. In the posterior space of the lower rectum, the presacral fascia and the fascia propria of the rectum define the supralevator space. In front of the rectum, Denonvilliers’ fascia and the fascia propria of anterior rectum define the prerectal space. These anatomical spaces are filled with loose connective tissue such as transversalis fascia and extraperitoneal fascia. The retrorectal space as well as the supralevator space behind the rectum and the prerectal space in front of the rectum are the optimal dissection planes for TME.

According to our previous study, the general anatomical structures of the abdomen could be divided into three layers and two interlayers. The abdominal and pelvic wall mainly played the role of support and protection. The urogenital system was located between transversalis fascia and extraperitoneal fascia, by which it was separated from the pelvic wall behind and from the rectum ahead, respectively (Figure 1A). The urogenital system layer was similar to an independent viaduct-like structure sandwiched between the pelvic wall and the rectum, and it gradually detached from the posterior pelvic cavity at the level of the S3/4 vertebrae and extended laterally and anteriorly to the anterior urogenital triangle (Figures 2–4).

Because of mutual compression between adjacent organs, the fascia propria of different pelvic anatomical layers occurred wherever they were closely attached to each other. The pelvic wall was separated from the urogenital system by the transversalis fascia, and when they were attached to each other, the presacral fascia of the pelvic wall layer and the posterior renal fascia (Zuckerkandl's fascia) of the urogenital system layer arose simultaneously (Figure 4). The presacral fascia and the posterior renal fascia defined the presacral space, which was occupied by the transversalis fascia (Figure 4). Similarly, the anterior renal fascia of the urogenital system layer and the fascia propria of the rectum arose simultaneously when these two layers were attached to each other. Toldt's fascia (the extraperitoneal fascia) occupied the retrorectal space, which was defined by the anterior renal fascia and the fascia propria of the rectum (Figure 4). The anterior renal fascia and the posterior renal fascia enclosed the urogenital system layer to form a urogenital–hypogastric sheath, carrying the hypogastric nerves, ureters, and genital vessels downward to the pelvis. Hence, the anterior renal fascia was continuous with Denonvilliers’ fascia in front of the rectum (Figure 4). Denonvilliers’ fascia and the fascia propria of anterior rectum defined the prerectal space, which was occupied by the extraperitoneal fascia (Figure 1B).

The lowest boundary of the urogenital system layer posterolateral to the mesorectum divided the rectum into the upper and lower sections (Figures 2–4). The transversalis fascia was continuous with the extraperitoneal fascia between the lowest boundary of urogenital system layer and fascia propria of the rectum, indicating that the urogenital system layer did not directly connect to the mesorectum, and the rectosacral fascia was the lower part of the urogenital system layer in the posterior pelvic cavity (Figures 2–4). Because there was no urogenital system layer behind the lower rectum, the presacral fascia and the fascia propria of the rectum constituted an apparent “holy plane”, called the supralevator space, which was a continuation of the presacral space (Figures 2E, 4). Within the supralevator space, the transversalis fascia constituted the “angel hair” composed of abundant loose connective tissues. The supralevator space behind the rectum was continuous with the prerectal space in front of the rectum and both were occupied by the extraperitoneal fascia (Figure 4).

### The correct surgical plane for mesenteric-based rectal surgery

According to the above intraoperative observation and schematic diagrams of the pelvis, it was clear that the retrorectal space was the correct surgical plane for mesenteric-based rectal surgery. This surgical plane was accessed by incision of the peritoneal reflection and separating the extraperitoneal fascia to mobilize the upper mesorectum, without damaging the prehypogastric nerve fascia and fascia propria of the rectum (Figures 2, 3).

The rectosacral fascia did not directly connect to the rectum. Therefore, the junction of the transversalis fascia and extraperitoneal fascia between the lowest boundary of the urogenital system layer and fascia propria of the rectum was severed to expose the holy plane. The angel hair within the holy plane was also separated to mobilize the lower mesorectum (Figures 2, 3). The holy plane, along with the transversalis fascia, extended laterally and anteriorly from the posterior pelvic cavity to the prerectal space. Therefore, the prerectal space was the correct plane for posterior mesenteric-based rectal surgery, without damage to Denonvilliers’ fascia and the fascia propria of the anterior rectum (Figure 4).

## Discussion

According to the peritoneum fusion theory, Toldt's fascia has been described as a fusion fascia formed by two layers of visceral peritoneum when the mesentery attaches to the posterior abdominal wall. On the one hand, Shinohara et al. indicated that as the organs contacted each other, the peritoneum dissolved and fused to form fusion fasciae, which became adherent and inseparable. Therefore, during surgical dissection, the correct plane should be within the loose connective tissue space between the fusion and subperitoneal fasciae to avoid damage to adjacent organs (16). Coffey et al. showed that there was a mesofascial plane between the deep mesothelium of the mesocolon and Toldt's fascia, and the mesofascial plane was the key to colorectal surgery (1). Mike et al. also believed that the interior of the fusion fascia could not be dissected (22). On the other hand, Gong et al. demonstrated that the fusion fascia, also called the secondary fascia or Toldt's fascia, could be dissected and surgeons could enter the loose fusion gap, which was the correct surgical plane (23). In addition, Liang et al. had shown that the correct plane for mobilization of the mesocolon was within Toldt's fascia (24). So, there are still some areas of confusion about the peritoneum fusion theory framework. We had previously proposed the peritoneum retreat theory, stating that when the mesocolon was attached to the adjacent organs, the visceral peritoneum of both would gradually retreat and bridge the gap between the surface of the bowel wall and adjacent organs, leaving the extraperitoneal fascia sandwiched between the mesocolon and adjacent organs (17). The extraperitoneal fasciae formed the optimal dissection plane for mesenteric-based surgery. Due to the removal of the confusion caused by fusion fasciae, the peritoneum retreat theory could greatly simplify the understanding of the abdominal fasciae and layers.

The best way to explore the anatomy and histology of the human body is based on its embryonic origin. However, there are still some difficulties in observing embryonic development due to ethical and technical factors. The anatomical structures of different adults should be naturally homologous and similar. A consensus about the anatomical structures of the pelvic cavity has not been reached because some anatomical layers might have been artificially created according to different subjective speculations.

Yang et al. reported that, in the retrorectal space, the urogenital–hypogastric sheath surrounded the fascia propria of the rectum posterolaterally and carried hypogastric nerves, ureter, and genital vessels downward to their terminations in the pelvis (25). Stelzner et al. also indicated that the parietal pelvic fascia was composed of inner and outer lamellae ensheathing the hypogastric nerves and ureter (12). The rectosacral fascia/rectosacral ligament were usually considered to originate from the parietal presacral fascia at the S3/4 vertebral level and join the rectal visceral fascia 3–5 cm above the anorectal junction, with the neurovascular bundles passing through the urogenital layer into the fascia propria of the rectum (12). The rectosacral fascia bridged the retrorectal and presacral space, inserted into the rectal wall and divided the retrorectal space into superior and inferior portions (9, 12). Kinugasa et al. showed that there was no fascial structure connecting directly between the fascia propria of the rectum and parietal presacral fascia (i.e., the rectosacral fascia) (7). Our study indicated that the urogenital–hypogastric sheath is actually a layer of the urogenital system that forms a sandwich-like, compound fascia sheath. This structure develops along with the fascia propria of the rectum and parietal presacral fascia due to mutual compression between these layers. We also showed that the rectosacral fascia was continuous with the urogenital system in the posterior pelvic cavity. The rectosacral fascia did not directly connect to the rectum, but was separated by the junction of the transversalis fascia and extraperitoneal fascia between the lowest boundary of the urogenital system layer and fascia propria of the rectum. Therefore, there were no neurovascular branches extending from the urogenital layer to the mesorectum, consistent with the research of Kinugasa et al. (7).

The anatomical segmentation of the rectum is still controversial. Usually, the rectum is equally divided into high, middle, and low segments, according to the distance from the anal margin. Alternatively, it can be divided into upper and lower halves by the anterior peritoneal reflection in front of the rectum, while the segmentation line behind the rectum remains unclear. In the posterior space of upper rectum, Stelzner et al. reported that the mesorectal fascia and inner lamella of the parietal pelvic fascia defined the retrorectal space, while the outer lamella of the parietal pelvic fascia and the presacral fascia defined the presacral space (12). In our study, a virtual line joined the superior margin of Denonvilliers’ fascia and the inferior margin of the rectosacral fascia at the S3/4 vertebral level and divided the rectum into upper and lower sections. We demonstrated that the posterior space of the upper rectum was divided into the retrorectal and presacral spaces by the independent urogenital system layer. The fascia propria of the rectum, anterior renal fascia, posterior renal fascia, and parietal presacral fascia simultaneously occur because of mutual compression between adjacent layers. The extraperitoneal fascia fills the retrorectal space between the fascia propria of the mesorectum and the anterior renal fascia, while the transversalis fascia fills the presacral space between the posterior renal fascia and the parietal presacral fascia.

In the posterior space of the lower rectum, previous studies have reported that the rectosacral fascia connects the parietal presacral fascia and fascia propria of the rectum, and divides the retrorectal space into inferior and superior portions (9, 12). Our study demonstrated that the posterior space of the lower rectum was below the rectosacral fascia and constituted an apparent holy plane by the presacral fascia and the fascia propria of the rectum. The extraperitoneal fascia consists of loose connective tissue, known as the angel hair, within the holy plane.

Some researchers have suggested that Denonvilliers’ fascia comprises two layers: the posterior layer is a direct extension of the proper rectal fascia, and the anterior layer is continuous with the presacral fascia (7, 10, 26). Other studies have found that Denonvilliers’ fascia is continuous with the inner lamella of the parietal pelvic fascia and extends from the peritoneal reflection toward the perineal body, separating the rectum from the genital organs (12, 27, 28). We showed that Denonvilliers’ fascia was continuous with the anterior renal fascia, and the prerectal space was continuous with the supralevator space. The prerectal space was confined by Denonvilliers’ fascia and the fascia propria of the anterior rectum and filled by the extraperitoneal fascia.

The fascia propria is a mesothelial cell layer that is crucial for mesenteric-based surgery, by helping to determine the correct surgical plane and ensure the integrity of the mesentery. There are four mesothelial layers around the rectum: the presacral fascia, posterior renal fascia, anterior renal fascia, and fascia propria of the rectum. Our previous studies have shown that there may be two means of mesenteric adhesion when the mesentery contacts adjacent organs: an antagonistic effect in thick areas and an adsorptive effect in thin areas (17, 19). On the one hand, these mesothelial layers are not congenitally present but rather are induced by mechanical stress due to the mutual compression of the adjacent organs in thick areas. On the other hand, adjacent tissues are absorbed and assimilated when they contact each other in areas with sparse blood vessels and thin layers of fat. Therefore, these mesothelial layers appear, disappear, and become intermittent in different regions.

It is not yet clear whether the lateral ligaments of the rectum exist (29–33). According to our previous studies, the three anatomical layers in the pelvic cavity are independent and adjacent tissues are absorbed and assimilated when they contact each other; hence, there is no evidence of the presence of lateral ligaments of the rectum. However, this mechanism requires confirmation through histological and cadaveric studies.

In summary, this study demonstrated the perirectal fascia and spaces according to the peritoneum retreat theory. Though this hypothesis might not be sufficiently validated and needed further verification, the peritoneum retreat theory could reasonably explain the formation process of abdominal fasciae and layers, helping surgeons better understand the surgical plane. The rectosacral fascia is a continuation of the urogenital system layer and is separated from the fascia propria of the rectum by the junction of the transversalis fascia and extraperitoneal fascia. The retrorectal space as well as the supralevator space behind the rectum and the prerectal space in front of the rectum are the optimal dissection planes for TME.

## Data Availability

The original contributions presented in the study are included in the article/Supplementary Material, further inquiries can be directed to the corresponding author/s.
